# Multiplex-PCR-Based Screening and Computational Modeling of Virulence Factors and T-Cell Mediated Immunity in *Helicobacter pylori* Infections for Accurate Clinical Diagnosis

**DOI:** 10.1371/journal.pone.0136212

**Published:** 2015-08-19

**Authors:** Sinem Oktem-Okullu, Arzu Tiftikci, Murat Saruc, Bahattin Cicek, Eser Vardareli, Nurdan Tozun, Tanil Kocagoz, Ugur Sezerman, Ahmet Sinan Yavuz, Ayca Sayi-Yazgan

**Affiliations:** 1 Department of Molecular Biology and Genetics, Istanbul Technical University, Maslak, Istanbul, Turkey; 2 Department of Medical Microbiology, School of Medicine, Acibadem University, Atasehir, Istanbul, Turkey; 3 Department of Internal Medicine, School of Medicine, Acibadem University, Atasehir, Istanbul, Turkey; 4 Department of Gastroenterology, Acibadem Hospital Group, Istanbul, Turkey; 5 Department of Biostatistics and Medical Informatics, School of Medicine, Acibadem University, Atasehir, Istanbul, Turkey; 6 Molecular Biology, Genetics and Bioengineering Program, Faculty of Engineering and Natural Sciences, Sabancı University, Tuzla, Istanbul, Turkey; The Ohio State University, UNITED STATES

## Abstract

The outcome of *H*. *pylori* infection is closely related with bacteria's virulence factors and host immune response. The association between T cells and *H*. *pylori* infection has been identified, but the effects of the nine major *H*. *pylori* specific virulence factors; *cagA*, *vacA*, *oipA*, *babA*, *hpaA*, *napA*, *dupA*, *ureA*, *ureB* on T cell response in *H*. *pylori* infected patients have not been fully elucidated. We developed a multiplex- PCR assay to detect nine *H*. *pylori* virulence genes with in a three PCR reactions. Also, the expression levels of Th1, Th17 and Treg cell specific cytokines and transcription factors were detected by using qRT-PCR assays. Furthermore, a novel expert derived model is developed to identify set of factors and rules that can distinguish the ulcer patients from gastritis patients. Within all virulence factors that we tested, we identified a correlation between the presence of *napA* virulence gene and ulcer disease as a first data. Additionally, a positive correlation between the *H*. *pylori dupA* virulence factor and IFN-γ, and *H*. *pylori babA* virulence factor and IL-17 was detected in gastritis and ulcer patients respectively. By using computer-based models, clinical outcomes of a patients infected with *H*. *pylori* can be predicted by screening the patient's *H*. *pylori vacA m1/m2*, *ureA* and *cagA* status and IFN-γ (Th1), IL-17 (Th17), and FOXP3 (Treg) expression levels. Herein, we report, for the first time, the relationship between *H*. *pylori* virulence factors and host immune responses for diagnostic prediction of gastric diseases using computer—based models.

## Introduction

*Helicobacter pylori (H*. *pylori)* is a gram negative, microaerophilic, spiral-shaped bacteria that colonize in human gastric mucosa and if not treated, it persists through lifespan. *H*. *pylori* infection has been implicated as the main cause of chronic gastritis, peptic ulcers and gastric adenocarcinoma [1, 2]. *H*. *pylori* is carried by more than half of the world’s population with prevalence as high as 90% in developing countries [3]. Although most infected individuals remain asymptomatic, 15–20% of *H*. *pylori* positive individuals develop at least one of the associated diseases at some point in their lives [4]. The clinical outcome of *H*. *pylori* infection is determined by multiple factors including *H*. *pylori* related virulence factors, host genetic predisposition and immune response [5, 6]. Many putative virulence genes have been described to determine the clinical outcome of *H*. *pylori* infection. In the recent studies, it is well established that virulence factors with potential value for specific pathologies are the cytotoxin associated gene A (*cagA*), vacuolating cytotoxin gene A (*vacA*), outer inflammatory protein A (*oipA*), the blood group antigen binding adhesin gene A (*babA*), the putative neuraminyllactose-binding hemagglutinin homolog (*hpaA*), neutrophil-activating protein A (*napA*), duodenal ulcer promoting gene A (*dupA*), urease A (*ureA*), and urease B (*ureB*) [7, 8]. Although virulence factors are important in conditioning the clinical outcome of the *H*. *pylori–*driven infection, the local immune response mechanisms have been claimed to play a role in the pathogenesis of the disease [9]. Regarding host immune response, CD4+ T cells including T- helper cells (Th) and regulatory T-cells (Tregs) are shown to play critical roles in the pathogenesis of *H*. *pylori* infection. T helper 1 (Th1) cells contribute to the host defense against pathogens by secreting various cytokines and through their effector functions. Recently described T helper 17 (Th17) cells are shown to play a role in host defense against *H*. *pylori* infection by mediating the recruitment of neutrophils and macrophages into infected tissues. *H*. *pylori* infection may induce a mixed Th1/Th17 response. Tregs have been identified as the major regulatory component of the adaptive immune response and they have been involved in *Helicobacter pylori*-related inflammation and bacterial persistence [10–14].

In previous studies, single or multiplex-PCR assays have been developed to detect the *H*. *pylori* virulence factors. However, these PCR assays are not sufficient to detect the most important *H*. *pylori* virulence factors simultaneously. Herein, we describe a sensitive and specific multiplex-PCR with a wide range of virulence factors which can detect nine virulence genes in three PCR reactions. This may help to better understand the correlation between the virulence factors and different clinical forms of gastric lesions and therefore the pathogenesis of each of these factors. Development of the gastric disease during *H*. *pylori* infection depends on an activated adaptive immune response controlled by T helper (Th) cells. However, the relative contributions of the Th1 and Th17 subsets and Tregs to gastric diseases and correlation of Th1, Th17 and Tregs with *H*. *pylori* virulence factors are not well understood. In this study, we investigate the association of the *H*. *pylori* virulence factors with Th1, Th17 and Treg cells by determining the cytokines and transcription factor specific for these T cells in mRNA expression levels. Herein this report, it is the first time that an expert derived model was developed to show the relationship between *H*. *pylori* virulence factors and host immune response for diagnostic prediction of gastric diseases.

## Materials and Methods

### Ethical Approval

Each participant included in this study provided a written informed consent to participate to the study. This study was approved by the ethical committee of the Acibadem University and Istanbul Technical University.

### Patients Selection

Biopsy specimens were obtained from the patients who underwent endoscope because of gastroduodenal diseases at the Gastroenterology Department of Acibadem Hospital Groups, in Istanbul, Turkey. In total, 80 patients were selected who fulfilled the following criteria for obtaining expulsion: under 18 years, 65 years or older patients with active infection, cancer patients, patients with inflammatory disease, patients who have had gastrointestinal bleeding within the last month and who have had previous gastrointestinal surgery, patients diagnosed with chronic liver failure, known renal failure, diabetic patients, pregnant women and patients who have previously received *H*. *pylori* eradication treatment, immunosuppressive therapy (including steroids), patients who have had NSAIDs and / or antibiotic treatment in the last three weeks, antisecretory therapy in the last two weeks and patients who refused to sign the informed consent form voluntarily. 18 *H*. *pylori* negative individuals were included to the study as a control group.

### Gastric biopsy specimens

Two gastric biopsy specimens were taken from the antrum and corpus part of the patients’ stomach during endoscopy, one for DNA isolation and the other for RNA isolation. Fresh biopsy specimens were placed into the RNAlater solution (Ambion, RNAlater RNA Stabilization Solution) and kept at + 4°C for overnight, then put at– 80°C deep freezer until DNA and RNA isolation.

### RNA extraction

To study gene expression, RNA was isolated from RNALater-stabilized human tissue specimens. Frozen biopsy specimens were homogenized by using a high efficiency homogenization system (SpeedMill PLUS, Analytikjena) and total RNA was isolated by using RNA isolation kit, following the protocol recommended by the manufacturer (innuSPEED tissue RNA kit Analytikjena). All RNA samples were quantified using NanoDrop (ND-2000, ROCHE) and maintained in– 80°C deep freezer until cDNA synthesis. cDNA was synthesized using 1 μg of RNA through a reverse transcription reaction (High capacity cDNA Reverse Transcription Kit, Applied Biosystems).

### DNA extraction

To study virulence factors of *H*. *pylori*, DNA was extracted immediately after RNA isolation by using a DNA isolation kit (Quick g-DNATM, ZYMO RESEARCH) following the manufacturer’s description. All DNA samples were quantified using NanoDrop (ND-2000, ROCHE) and maintained in– 80°C deep freezer.

### Primer design

Primers, obtained from metabion international AG, Germany, were used in this study. For the PCR assay primers, GenBank entries were searched for the selected virulence genes sequences including *ureA*, *ureB*, *cagA*, *oipA*, *hpaA*, *babA*, *dupA*, *and napA*. The primers were designed by using Primer 3 software (Table 1). For amplification of *vacA s1/s2*, *vacA m1/m2* genes previously published PCR primers (Table 1) were used [15–18]. For the quantitative RT-PCR assay primers, GenBank entries were searched for sequences of the genes encoding human IFN-γ, T-bet, IL-17, RORγt, FOXP3. The published sequences were aligned and primers were designed by using the LightCycler probe design software (Roche Diagnostics, Mannheim, Germany) (Table 2). A BLAST search was performed to confirm the specificity of the DNAsequences of all the primers (http://www.ncbi.nlm.nih.gov/BLAST/).

**Table 1:** Multiplex-PCR primers designed for the amplification of *H*. *pylori* virulence genes.

DNA region(s) amplified	Primer Name	Sequences (5′→3) PCR	Product Size (bp)	References
*ure A*	ure A-F	TGATGGGACCAACTCGTAACCGT	244	This study
*ure A*	ure A-F	CGCAATGTCTAAGCGTTTGCCGAA	244	This study
*ure B*	ure B-F	AGTAGCCCGGTGAACACAACATCCT	645	This study
*ure B*	ure B-F	ATGCCTTTGTCATAAGCCGCTTGG	645	This study
*cag A*	cag A-F	AGAGCAAGCGTTAGCCGATCTCAA	415	This study
*cag A*	cag A-R	TTTCCCTACACCACCCAAACCACT	415	This study
*hpa A*	hpa A-F	TAGTGGGATGCAGCCCGCATATTA	534	This study
*hpa A*	hpa A-R	CGCTATGGCTTGAATGGGTGGTTT	534	This study
*oip A*	oip A-F	GTTTTTGATGCATGGGATTT	401	[15]
*oip A*	oip A-R	GTGCATCTCTTATGGCTTT	401	[15]
*bab A*	bab A-F	AATCCAAAAAGGAGAAAAAGTATGAAA	832/601	[16, 17]
*bab A*	bab A-R	TGTTAGTGATTTCGGTGTAGGACA	832/601	[16, 17]
*dup A*	dup A-F	TGAGCGTGGTAGCTCTTGAC	584	This study
*dup A*	dup A-R	GAGCGCGTTAGCGATATAGG	584	This study
*nap A*	nap A-F	GAATGTGAAAGGCACCGATT	304	This study
*nap A*	nap A-R	ATCGTCCGCATAAGTTACGG	304	This study
*vac A s1/s2*	vac A s1/s2-F	ATGGAAATACAACAAACACAC	259/286	[1]
*vac A s1/s2*	vac A s1/s2-R	CTGCTTGAATGCGCCAAAC	259/286	[1]
*vac A m1/m2*	vac A m1/m2-F	CAATCTGTCCAATCAAGCGAG	567/642	[18]
*vac A m1/m2*	vac A m1/m2-R	GCG TCTAAATAATTCCAAGG	567/642	[18]

**Table 2:** Quantitative RT-PCR primers designed for the detection of Th1, Th17 and Treg cell response.

Primer Name	Sequences (5′→3)	References
IL-17-F	CCTGGGAAGACCTCATTGGT	This study
IL-17-R	ATTCCAAGGTGAGGTGGATCG	This study
RORγt-F	CTGCAAAGAAGACCCACACC	This study
RORγt-R	GCAGTTCTGCTGACGGGT	This study
IFN-γ-F	TCCAAAAGAGTGTGGAGACCA	This study
IFN-γ-R	TCGACCTCGAAACAGCATCT	This study
FOXP3-F	TGACAGTTTCCCACAAGCCA	This study
FOXP3-R	GAAGATCTCGGCCCTGGAAG	This study

### Multiplex-PCR assay

Genomic DNA isolated from *H*. *pylori* G27 strain used in this study were a kind gift from Prof. Dr. Anne Mueller from University of Zurich, Institute of Molecular Cancer Research, Switzerland. Genomic DNA of *H*. *pylori* G27 strain and gastric specimens were used as target DNAs in multiplex-PCR assays. The amplification reactions were carried out in a total volume of 25 μl and the multiplex-PCR reaction mixture consisted of 0.65 U of Dream Taq DNA polymerase (Thermo Scientific), 2.5 μl from 10X DreamTaq Buffer (includes 20 mM MgCl2), 20 μM of forward and reverse primers, 200 μM of each dNTP and 3 μl DNA. Amplification programme included an initial denaturation step at 95°C for 3 min followed by 45 cycles of denaturation at 95°C for 45 s, primer annealing at 60°C for 45 s and primer extension at 72°C for 2 mins, with final extension step at 72°C for 5 mins. The PCR products were subjected to electrophoresis on agarose gels and stained with SYBR Gold (Invitrogene). The specificity of the primer pairs was confirmed by employing a positive control.

### Quantitative RT-PCR assay

Optimal annealing temperatures of the qRT-PCR primer pairs and expected product size were determined with conventional RT-PCR. SYBR Green qRT-PCR amplifications were performed using a LightCycler 480 Real-Time Detection System (ROCHE). All qRT-PCR experiments were carried out in duplicate with a reaction volume of 10 μl, using 96-well optical grade PCR plates (ROCHE) covered with optical-quality sealing film (ROCHE). The efficiency of qRT-PCR amplification was optimized for each primer pair, using various dilution series of cDNA. The reaction mixtures for the optimized LightCycler 480 SYBR Green I Master Mix based assays consisted of 5μl SYBR Green I Master Mix, 0.5 μM from each primer, 1.5 μl PCR grade water and 2.5 μl target cDNA. The amplification reaction was performed with preliminary denaturation for 10 min at 95°C, followed by 40 amplification cycles of denaturation at 95°C for 15 s, primer extension at 58°C for 1 min, extension at 72°C for 1, 30 min and additional final extension at 72°C 5 min. A final cooling step was performed at 4°C. The percentage of expression of the cytokines and the transcription factors are calculated from quantitative RT-PCR results based on the cycle threshold (Ct). Relative quantification method was used to analyze the PCR. Relative quantification describes the change in expression of the target gene relative to the Hp negative control group. 2^-^ΔΔCT Method is used to calculate relative changes in the gene expression determined by the quantitative RT-PCR and as an internal control 18S rRNA housekeeping gene primers were used to normalize the quantitative RT-PCR. Negative controls without cDNA and positive controls for gene were included

### Statistical analysis

A two-tailed Fisher’s exact test was performed to examine the relationship between *H*. *pylori* virulence factors and gastric disease type (i.e. Gastritis or Ulcer). Additionally, correspondence between virulence factor presence and immune response was assessed with Pearson product-moment correlation coefficients. Statistical significance of correlations between virulence factors and immune response factors was assessed using a t-test. All calculations were performed using R (version 3.1.0, The R Foundation for Statistical Computing, Vienna, Austria; http://www.r-project.org).

### Expert-derived models for diagnostic prediction

Classification of samples into gastritis and ulcer classes was performed using expert-derived model. The model was built on a randomly selected training data, which was consisted of two-thirds of the all dataset. Model building was performed by determining the most important feature first that can distinguish the ulcer and gastritis patients, and adding other features if they improve the overall accuracy of the prediction. Then best model was selected and validated on a test set. Remaining one third of the data was randomly selected as testing set, and this process was repeated for 1000 times. Classification performance was assessed using prediction accuracy for each class. Mean of accuracies and their standard deviations were reported. All calculations were performed using R (version 3.1.0, The R Foundation for Statistical Computing, Vienna, Austria; http://www.r-project.org).

## Results

### Detection of *H*. *pylori* virulence factors by multiplex-PCR

**Fig 1:**
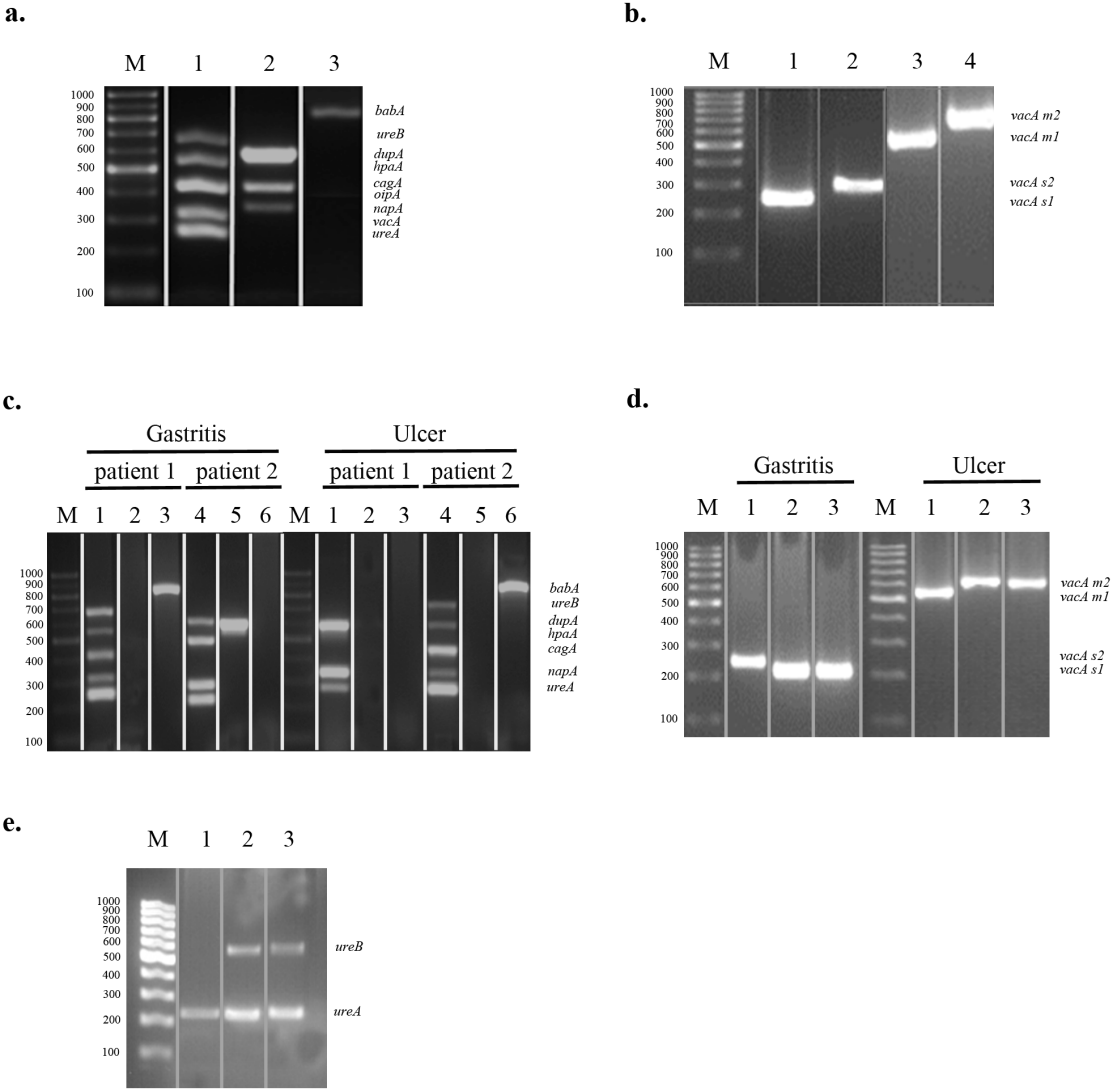
Multiplex-PCR Assay For *Helicobacter*-specific Virulence Factors. **(a)** amplification of virulence genes of *H*. *pylori* G27 strain by multiplex PCR; Lane M, 100 bp- marker (ThermoSCIENTIFIC, Gene Ruler), Lane 1 (Reaction 1); *ureA* (244bp), *ureB* (645bp), *hpaA* (534bp), *cagA* (415bp), *napA* (384bp), Lane 2 (Reaction 2); *dupA* (584bp), *oipA* (401bp), *vacA* (333bp), and Lane 3 (Reaction 3); *babA* (832bp) **(b)** PCR inferred results of *s1/s2* and *m1/m2* allelles of *vacA* gene; Lane M, 100 bp- marker (ThermoSCIENTIFIC, Gene Ruler), Lane 1 (reaction 1); *vacA s1* (259bp), Lane 2 (Reaction 2); *vacA s2* (286 bp), Lane 3 (Reaction 3); *vacA m1* (567 bp), and Lane 4 (Reaction 4) *vacA m2*(642 bp). **(c)** Multiplex-PCR application of the biopsy samples taken from randomly selected patients with ulcer and gastritis; Lane M, 100 bp- marker (ThermoSCIENTIFIC, Gene Ruler), between Lane 1 to 6 are representing patients with gastritis or ulcer: Multiplex PCR reaction results of patient 1 and 2 (gastritis or ulcer) are shown between Lane 1 to 3 and Lane 4 to 6, respectively. Reaction 1 (Lane 1 and 4) *ureA* (244bp), *ureB* (645bp), *hpaA* (534bp), *cagA* (415bp), *napA* (384bp), Reaction 2 (Lane 2 and 5); *dupA* (584bp), *oipA* (401bp), *vacA* (333bp), and Reaction 3 (Lane 3 and 6); *babA*(832bp) **(d)** PCR inferred results of *s1/s2* and *m1/m2* allelles of *vacA* gene of randomly selected patients with gastritis and ulcer respectively; Lane M, 100 bp- marker (ThermoSCIENTIFIC, Gene Ruler), between Lane 1 to 3 are representing patients with gastritis (left side) or ulcer (right side): Patients with gastritis; Lane 1–3, *vacA s1* (259bp), and *vacA s2* (286 bp), patients with ulcer; Lane 1–3, *vacA m1* (567 bp), and *vacA m2* (642 bp). **(e)** Multiplex urease-PCR assay to detect the *Helicobacter* positive and negative samples; Lane M, 100 bp- marker (ThermoSCIENTIFIC, Gene Ruler), Lane 1–2 are from randomly selected patients, and lane 3 is from *H*.*pylori* positive control strain G27. Sybr Gold (Invitrogene) was used for the gel in (a) and (c) and gel pictures were taken by using Observable Real Time Gel Electrophoresis System (Salubris Technica, Turkey).

Initially, to optimize the concentrations of the conventional multiplex-PCR components, the specificity of each primer pair, and the thermocycling parameters for each virulence factor, genomic DNA isolated from *H*. *pylori* G27 strain were used as the target DNA. We optimized multiplex-PCR of genomic DNA derived from *H*. *pylori* G27 strain to detect nine *H*. *pylori* virulence genes within a three PCR reaction; *ureB*, *hpaA*, *cagA*, *napA*, *ureA* in a single reaction, *dupA*, *oipA*, *vacA* in a single reaction, and *babA* in a single reaction (Fig 1(a) and 1(b)). Next, the multiplex-PCR was applied to genomic DNA derived from total of 80 *H*. *pylori* positive patients; 18 with ulcer and 62 with gastritis. Two representative multiplex-PCR data of nine *H*. *pylori* virulence genes obtained from these gastric specimens are shown in Fig 1(c). In order to investigate *H*. *pylori vacA* subtypes in *vacA* positive gastric specimens, we performed four separate PCR reactions (Fig 1(d)). The genes encoding *H*. *pylori* urease which are *ureA* and *ureB*, were also detected by a single multiplex-urease PCR assay to confirm the presence of *H*. *pylori* in gastric biopsy specimens which were detected by rapid urease test and pathological staining following endoscopy (Fig 1(e)). Hp-uninfected control group was used to check the multiplex-PCR primers for the virulence genes to monitor non-specific amplicons.

### Correlation between the manifestations of gastric disease and bacterial virulence factors

Based on multiplex-PCR results which were performed using 18 patients with ulcer and 62 patients with gastritis, no statistically significant difference was detected for the existence of *H*. *pylori cagA*, *hpaA*, *oipA*, *babA*, *vacAs1*, *vacAs2*, *vacAm1*, *vacAm2*, *ureA*, *ureB a*nd *dupA* virulence factors among patients with gastritis and ulcer (Table 3). *H*. *pylori cagA* gene amplification was found in 60% of all isolates; 61% of patients with ulcer and 60% of patients with gastritis. The *H*. *pylori vacA s1/m2* genotypes were the most frequent allelic combination of the vacA gene detected both among gastritis and ulcer patients. The *H*. *pylori oipA* gene prevalence was more frequent in ulcer patients than that of gastritis patients (50%, 35.48%). Also, there were no statistically significant difference between the patients with ulcer and gastritis for the presence of *H*. *pylori hpaA* gene (88.89% in ulcer patients, 74.19% for in gastritis patients), for *babA* gene (22.22% in ulcer patients, 14.52% for in gastritis patients), for *ureA* gene (100% in ulcer patients, 93.55% for in gastritis patients) and for *ureB* gene (66.67% in ulcer patients, 70.97% for in gastritis patients). Additionally, no ulcer patients were positive for dupA and only 8.06% of gastritis patients were positive for dupA gene.

**Table 3:** Comparison of virulence genes of *H*. *pylori* strains isolated from patients suffering from gastritis and ulcer.

Virulence Genes Characteristic		Strain Type				
		Ulcer		Gastritis		
		n	%	n	%	P-value
*cagA*						
	Absent	7	38.89	25	40.32	1.000
	Present	11	61.11	37	59.68	
*hpaA*						
	Absent	2	11.11	16	25.81	0.335
	Present	16	88.89	46	74.19	
*oipA*						
	Absent	9	50.00	40	64.52	0.285
	Present	9	50.00	22	35.48	
*babA*						
	Absent	14	77.78	55	85.48	0.475
	Present	4	22.22	7	14.52	
*vacA s1*						
	Absent	8	44.44	31	50.00	0.791
	Present	10	55.56	31	50.00	
*vacA s2*						
	Absent	15	83.33	55	88.71	0.686
	Present	3	16.67	7	11.29	
*vacA m1*						
	Absent	11	61.11	49	79.03	0.134
	Present	7	38.89	13	20.97	
*vacA m2*						
	Absent	9	50.00	17	27.42	0.090
	Present	9	50.00	45	72.58	
*ureA*						
	Absent	0	0.00	4	6.45	0.570
	Present	18	100.00	58	93.55	
*ureB*						
	Absent	6	33.33	18	29.03	0.774
	Present	12	66.67	44	70.97	
*dupA*						
	Absent	18	100.00	57	91.94	0.582
	Present	0	0.00	5	8.06	
*napA*						
	Absent	8	44.44	49	79.03	0.007*
	Present	10	55.56	13	20.97	

*H*. *pylori* strains with *napA* virulence factor are found more frequently in patients with ulcer than gastritis. Statistical significance was assessed with two-sided Fisher’s exact test.

However, the prevalence of *H*. *pylori napA* virulence factor was significantly higher in patients with ulcer than gastritis (Table 3).

### Correlation between *H*. *pylori* virulence factors and involvement of Th1, Th17 and Treg in H. pylori-induced gastric diseases

To investigate whether there are associations between *H*. *pylori* virulence factors and Th1, Th17, and Treg cell responses, we examined the expression levels of IL-17, RORγt, FOXP3, and IFN-γ in human gastric tissue specimens by using quantitative RT-PCR (qRT-PCR). Based on qRT-PCR results, *H*. *pylori* infected ulcer and gastritis patients were shown mainly to have a Th17 response instead of Th1 and Treg response. 88% Th17, 33% Th1 and 5% Treg responses were observed in patients with ulcer and 80% Th17, 35% Th1 and 0% Treg responses were observed in patients with gastritis. Then we assessed the relationship between our findings and the virulence factors by using Pearson’s correlation method (Table 4). 18 *H*. *pylori* negative individuals were used as a control group to verify the Th1 and Th17 immune response differences between the Hp-infected and Hp-uninfected patients, and in order to normalize our method in quantitative RT-PCR. Our data demonstrated a positive correlation between the *H*. *pylori dupA* virulence factor and IFN-γ in gastritis patients (r = 0.31, N = 62, p<0.05).

**Table 4:** Correlation between virulence factors and cytokines, transcription factors.

Virulence Factors/ Cytokines and Transcription Factors	Gastritis				Ulcer			
	IL-17	FOXP3	IFN-γ	RORγt	IL-17	FOXP3	IFN-γ	RORγt
*cagA*	0.21	-0.05	0.05	-0.02	0.17	-0.31	0.14	-0.34
*hpaA*	0.02	-0.02	-0.01	-0.09	0.27	0.04	0.15	0.17
*oipA*	-0.18	-0.09	0.07	0.06	-0.23	0.27	0.27	0.27
*babA*	-0.16	0.13	-0.13	-0.08	**0.74**	-0.11	-0.01	-0.15
*vacA s1*	0.13	-0.08	0.17	0.19	0.11	0.16	0.14	-0.16
*vacA s2*	0.02	0.09	-0.02	-0.08	-0.01	-0.08	-0.09	0.42
*vacA m1*	0.09	0.00	0.09	0.17	0.03	0.36	-0.14	0.28
*vacA m2*	-0.13	0.07	-0.06	-0.12	0.08	-0.26	0.22	-0.16
*ureA*	-0.15	-0.17	-0.01	-0.33	NA	NA	NA	NA
*ureB*	-0.12	0.02	0.05	-0.09	-0,20	0.10	-0.06	-0.40
*dupA*	-0.18	0.01	**0.31**	-0.04	NA	NA	NA	NA
*napA*	-0.07	0.02	-0.04	0.09	0.05	0.13	-0.38	0.10

A Pearson’s correlation between virulence factors and cytokines were calculated using R (version 3.1.0, The R Foundation for Statistical Computing, Vienna, Austria; http://www.r-project.org).

Additionally, IL-17 gene expression was shown for the first time to significantly positively correlated with the *H*. *pylori babA* virulence factor in ulcer patients (r = 0.74, N = 18, p<0.001).

### Expert-derived models for diagnostic prediction of gastric diseases using *H*. *pylori* virulence factors and host immune responses

**Fig 2:**
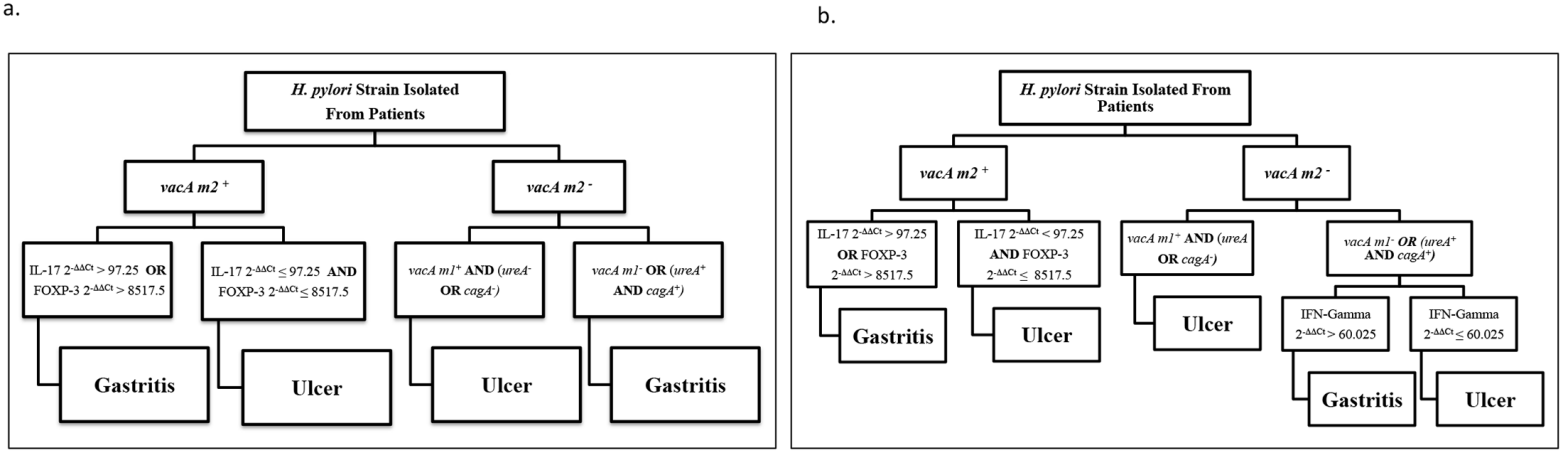
Expert-derived models for diagnostic prediction of gastric diseases using *H*. *pylori* virulence factors and host immune responses. **(a)** shows that a possible relationship prediction between the *vacAm1/m2*, *cagA* and *ureA* and IL-17, FOXP3 and patient’s clinical outcomes and **(b)** shows that the possible relationship prediction between *vacAm1/m2*, *cagA* and *ureA* and IL-17, FOXP3 and IFN-γ and patient’s clinical outcomes.

We created two expert-derived models (Fig 2(a) and 2(b)), which show the relationship between the *H*. *pylori* specific virulence factors and Th1, Th17 and Treg response on the basis of their specific cytokines and transcription factor levels in *H*. *pylori* infected gastritis or ulcer patients. By the aid of expert-derived models, knowledge of *H*. *pylori* virulence factors: *vacAm1/m2*, *cagA* and *ureA* and IL- 17, FOXP3 and IFN-γ expression level, it is possible to predict a patient’s clinical outcomes.

Performance evaluation using repeated test set sampling showed that first model (Fig 2(a)) has mean accuracy of 79% (standard deviation: 9%) in gastritis classification, 44% (standard deviation: 16%) in ulcer classification. Overall mean classification accuracy of this model was found as 69% (standard deviation: 8%). On the other hand, performance evaluations of the second model (Fig 2(b)) with repeated test set sampling resulted in a performance of 71% (standard deviation: 1%) mean accuracy for gastritis predictions, 61% mean accuracy (standard deviation: 17%) for ulcer predictions, which corresponds to a 68% overall mean accuracy (standard deviation: 9%). Confusion matrix of models for best performing test set samples can be found in Table 5.

**Table 5:** Confusion matrices for test set predictions.

Based on Diagnosis/ Based on Model	First Model		Second Model	
	Actual Gastritis	Actual Ulcer	Actual Gastritis	Actual Ulcer
**Predicted Gastritis**	14	0	13	1
**Predicted Ulcer**	2	4	0	6

Test set samples with best overall classification accuracy was used in calculation of confusion matrices. The actual numbers were the numbers that represent the patients with gastritis and ulcer diagnosed based on clinical diagnosis. The predicted numbers were the number of patients with ulcer or gastritis based by the models.

## Discussion

The virulence factors of *H*. *pylori*, which are known to be directly correlated with the extreme degree of genetic heterogeneity in *H*. *pylori* genomes, play a pivotal role in determining the outcome of *H*. *pylori* infection. The immune response of the host including T cell activation has also been the subject of recent studies, together with specific virulence factors of *H*. *pylori* [19]. Multiplex-PCR based genotyping systems have initially been developed to detect the *Helicobacter pylori*-specific virulence factors. However, these assays were not sufficient to detect many *Helicobacter pylori*-specific virulence factors simultaneously that are thought to be associated with bacterial pathogenesis and increase the risk for developing severe clinical manifestations. In our present study, a genotyping system-based multiplex-PCR assay was developed to detect nine potential virulence genes (*vacA*, *cagA*, *oipA*, *babA*, *hpaA*, *dupA*, *napA*, *ureA*, *ureB*) within three PCR reactions directly from human gastric biopsies. This assay is able to detect both the presence and absence of *H*. *pylori* by *ureA* and *ureB* genes, and identify the leading disease-associated virulence genes of *H*. *pylori* strains isolated from patients. Moreover, this PCR assay helps to better understand the correlation between the virulence factors and different clinical forms of gastric lesions and the pathogenesis of each of these factors.

Our multiplex-PCR assay results indicated no correlation between the *H*. *pylori* genes *vacA*, *cagA*, *oipA*, *babA*, *hpaA*, *dupA*, *ureA*, *ureB* and *H*. *pylori*-related gastritis and ulcer diseases. However, we identified a statistically significant correlation (p = 0.007) between *napA* gene and *H*. *pylori*-associated ulcer disease. In the literature it was indicated that activation of neutrophil may contribute to the damage of stomach [20]. However, we could not find any research like in our study in which the relationship between the *napA* gene and ulcer disease was showed clearly. It might be the first study to indicate the correlation between *H*. *pylori* related-ulcer disease and presence of *napA* virulence factor gene.

The type of host immune response, particularly driven by T cells, is crucial for the outcome of *H*. *pylori* infection in humans. Given the increasing number of reports, correlations between the characteristics of the T helper cell responses, including Th1, Th17, and Treg cell response and virulence factors should be of great interest in determining the outcomes of *H*. *pylori* infections. In previous studies, it was shown that Th1 cells contributed to inflammation and participated in the pathogenesis of *H*. *pylori* infection. However, the role of Th17 has not been clearly explicated. The efficacy of Th17 for the host protection against Gram-negative bacteria has also been shown [13]. In this study, we characterized the cell responses of T helper cells, particularly Th1 and Th17, in clinical isolates of *H*. *pylori* obtained from patients with gastritis and ulcer. One of the most prominent outcomes of the study is the identification of a higher Th17 cell response compared to Th1 response to *H*. *pylori* infection in patients with gastritis and ulcer. This finding has been shown previously in mice models [21], while this is the first time that it is identified in clinical samples. A possible reason for this outcome is that Th17 cells are clearly implicated in the pathogenesis of *H*. *pylori* infection by promoting the mucosal infection and contributing to bacterial colonization. Also Th1 cells cause mucosal inflammation in *H*. *pylori* infection, but our results suggested that Th17 cell responses might be induced earlier than Th1 cell responses in *H*. *pylori* infected ulcer and gastritis patients.

The *H*. *pylori* specific virulence factors are important for the pathogenesis of bacterium. These virulence factors facilitate the survival of the bacterium and immune response. In support of this, we found that there was a correlation between *dupA* positive strains and IFN- γ (Th1-specific cytokine) expression level in patients with gastritis. Our results confirmed a previous study that was indicated that *dupA* causes gastric inflammation and pathology either directly or indirectly over action upon infiltrating leukocytes rather than upon epithelial cells. It was also referred in the study that *dupA* positive *H*. *pylori* strains increase the risk of disease by stimulating a more definite Th1 response [22]. *babA* virulence factor helps bacteria to adhere to the fucosylated Lewis b antigen on the surface of the gastric epithelial cells. *H*. *pylori* adheres to the host and secretes effector molecules that can change function and viability of gastric epithelial cells. The production of various cytokines, gastric inflammation, and epithelial cell damage is enhanced by these changes. Also recent studies have indicated that there is severe mucosal injury in patients infected with *babA* positive *H*. *pylori* strains [23]. Our data showed for the first time that there is a positive relationship between *babA* positivity and interleukin-17 (IL-17) expression levels in patients with ulcer.

In this present study, we identified significant correlations between the virulence factors of *H*. *pylori*, responses of T cells (Th1, Th17, Treg) and clinical outcomes of *H*. *pylori* infections, and these eventually helped evolution of expert-derived models. Initially, first model (Fig 2(a)) was developed using the strategy outlined in the method section; however, low ulcer classification accuracy of this model indicated that there is a need for additional factors to distinguish ulcer and gastritis patients more accurately. With this motivation, in transition to model 1 (Fig 2(a)) to model 2 (Fig 2(b)), we have used an additional factor (IFN- γ) that eventually improved the ulcer classification accuracy from 44% to 61% with a cost of only one misclassified gastritis patient. Further more in the first model predictions were done by using the results of the relationship between the virulence factors, Th1 and Treg response while in the second model Th1 response also was added to these combinations. To our knowledge, this is the first report of expert-derived models that are based on the relationship between *H*. *pylori* virulence factors and host immune responses. Using the proposed models, the likelihood for a patient to have gastritis or ulcer can be estimated by screening the presence of *H*. *pylori* virulence genes (i.e. *vacA m1/m2*, *ureA* and *cagA*) and expression levels of IL-17, FOXP3 and IFN- γ in the host. In the future, the model can be developed further to be utilized as a supporting tool in the diagnostic prediction of life- threatening gastric diseases, such as ulcer.

In conclusion, our current data support the hypothesis that the relationships between the *H*. *pylori* specific virulence factors and Th1, Th17 and Treg cells have an important role in the development of *H*. *pylori* infection and help to estimate the clinical outcomes of *H*. *pylori* infection. A better understanding of the relationship between virulence factors and T cell responses may help to guess the clinical outcomes of *H*. *pylori* infection and prevents risk for gastric cancer development.
